# Eruptive disseminated porokeratosis with diagnostic dermoscopic clues

**DOI:** 10.1016/j.jdcr.2025.09.010

**Published:** 2025-09-19

**Authors:** Claudia Liliana Montoya Maya, Juan Camilo Marchán Cárdenas, Jairo Francisco Fuentes Carrascal, Esperanza Meléndez Ramírez, Beatriz Elena Orozco Sebá, Jesús Pérez García

**Affiliations:** a Dermatologist, Hospital Universidad del Norte, Professor, Universidad del Norte, Barranquilla, Colombia; b PGY-2 Dermatology Resident, Hospital Universidad del Norte / Universidad del Norte, Barranquilla, Colombia; c Pathologist, Professor of Dermatology Postgraduate Program, Universidad del Norte, Barranquilla, Colombia

**Keywords:** cornoid lamella, dermoscopic features, eruptive disseminated porokeratosis, keratin rim, porokeratosis

## Clinical presentation

**Fig 1:**
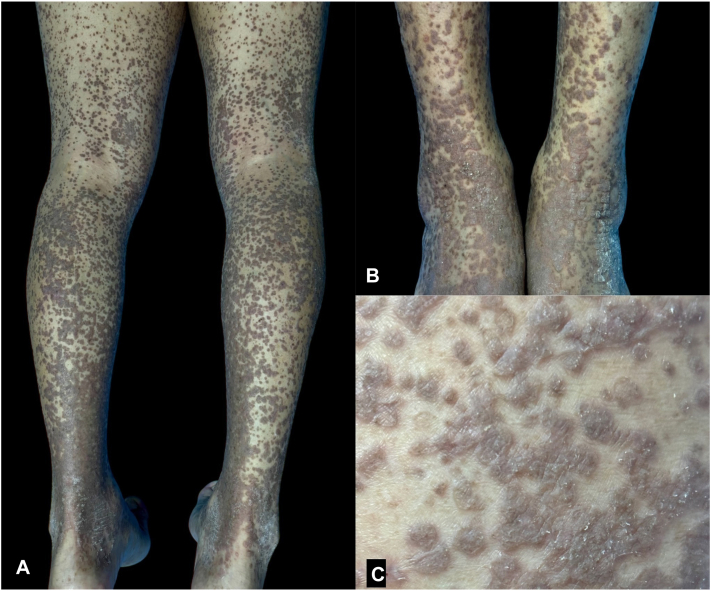
**A,** Multiple well-demarcated *brown papules* with a rough, scaly surface coalescing into extensive plaques on the lower extremities. **B,** Confluence of papules forming infiltrated plaques extending to the dorsal feet. **C,** Closer view of the papules, showing sharp borders and overlying fine scale.

A 73-year-old man presented with a 15-month history of a pruritic eruption that began as brown papules on the right thigh. The lesions rapidly disseminated to the bilateral lower extremities within 2 months. He was initially treated for hypertrophic lichen planus and later for Grover’s disease, receiving topical corticosteroids and narrowband UV-B phototherapy without response. Physical examination revealed numerous coalescing, well-demarcated brown papules and plaques with a rough, scaly surface, distributed symmetrically from the pelvis to the dorsal feet (Fig 1).

## Dermoscopic appearance

**Fig 2:**
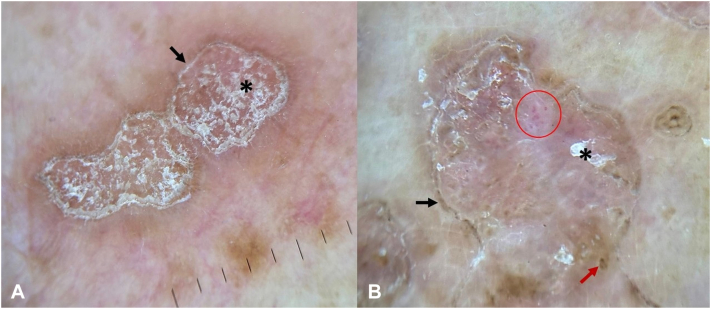
**A, B,** Dermoscopic images showing a sharply demarcated keratin rim (*black arrows*), central scale (*asterisk*), peripheral *brown dots* (*red arrow*), and focal glomerular vessels (*red circle*).

Dermoscopy revealed a sharply demarcated keratin rim with peripheral brown dots, central scale, and focal glomerular vessels (Fig 2). In a nonspecific pruritic eruption with multiple prior differentials, dermoscopy was the first modality to raise suspicion of porokeratosis.

## Histologic diagnosis

**Fig 3:**
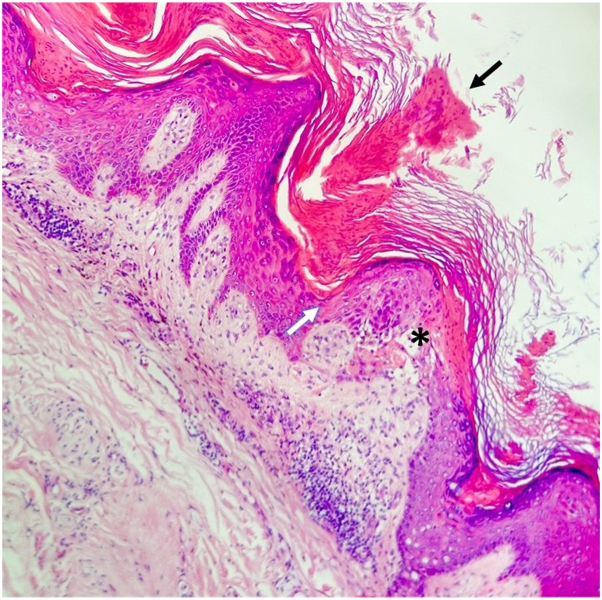
Hematoxylin and eosin stain, 10×. A cornoid lamella, represented by a vertical column of parakeratosis (*black arrow*), overlying a focus of hypogranulosis (*white arrow*) with underlying vacuolar interface changes (*asterisk*).

Histopathology revealed a vertical column of parakeratosis resting on focal hypogranulosis and vacuolar interface changes (Fig 3), consistent with a cornoid lamella—a finding that correlates with the keratin rim observed on dermoscopy. Based on clinical distribution and onset, the case was classified as eruptive disseminated porokeratosis.

Key message

Eruptive disseminated porokeratosis is a rare variant with variable clinical morphology, often delaying recognition. Dermoscopy was essential in this case, revealing a classic keratin rim with peripheral pigmentation and glomerular vessels. While these features are shared across porokeratosis subtypes, dermoscopic documentation of this variant remains limited.^1^ Early recognition may guide timely biopsy and management, given its potential association with malignancy^2^; in this patient, active screening revealed no underlying neoplasms.

## Conflicts of interest

None disclosed.
